# Polyurethane Glycerolysate as a Modifier of the Properties of Natural Rubber Mixtures and Vulcanizates

**DOI:** 10.3390/ma17010062

**Published:** 2023-12-22

**Authors:** Marcin Włoch, Maksymilian Toruńczak, Janusz Datta

**Affiliations:** Department of Polymers Technology, Faculty of Chemistry, Gdańsk University of Technology, G. Narutowicza Str. 11/12, 80-233 Gdańsk, Poland; s165836@student.pg.edu.pl

**Keywords:** natural rubber, chemical recycling, polyurethane glycerolysate, modifier, vulcanization kinetics, mechanical properties, thermo-mechanical properties, thermal stability

## Abstract

Chemical recycling of polyurethanes can be realized in several different ways, but the most important methods are glycolysis and glycerolysis. Both methods permit recovery of polyols (when the process is realized with the mass excess of depolymerizing agent) or substitutes of polyols, which contain urethane moieties in the main chains and terminate mainly in hydroxyl groups (when the process is realized with the mass excess of depolymerized polyurethane). Oligomeric products with urethane groups in the chemical structure can also be used as modifiers of rubber mixtures and vulcanizates. The main aim of the presented work is to study the effect of polyurethane glycerolysate on the performance of natural rubber mixtures and vulcanizates. The influence of the modifier on the vulcanization kinetics and swelling of rubber mixtures, and the thermo-mechanical and mechanical properties of rubber vulcanizates, was studied. The prepared materials were also subjected to accelerated thermal aging in air. It was found that polyurethane glycerolysate affects the vulcanization process of rubber mixtures (for example, promotes the activation of vulcanization) and acts as an antidegradant under thermoxidative conditions (higher stability of mechanical properties was observed in comparison to a reference sample without modifier). The obtained results show that chemical recycling products can be valuable modifiers of natural rubber mixtures and vulcanizates, which extends the possible applications of polyurethane chemical recycling products.

## 1. Introduction

The chemical recycling of polyurethanes involves several different methods, i.e., glycolysis [1,2,3,4,5], glycerolysis [6,7,8,9], acidolysis [10,11,12], aminolysis [13,14], phosphorolysis [15,16,17,18], and others. The process most described in the literature is glycolysis, which is realized using low-molecular weight diols, e.g., 1,2-ethanediol, 1,3-propanediol, 1,4-butanediol, 1,5-pentanediol, and 1,6-hexanediol. Glycolysis can be realized with mass excess of depolymerizing agent or with the mass excess of depolymerized polyurethane. The first method, realized with the mass excess of low-molecular weight diol to depolymerized polyurethanes (also called split-phase glycolysis), results in the recovery of polyol that was used in the synthesis of depolymerized polyurethane. The products obtained by this method mainly consist of two or more phases, and one of them is mainly composed of the polyol and should be purified before further use in the synthesis of new polyurethanes [19]. The second method, which is realized with the mass excess of depolymerized polyurethane, mainly results in the formation of one-phase product that can be used as a polyol substitute (polyol containing urethane moieties in the main chain and terminating in hydroxyl groups) in the synthesis of new polyurethanes [4]. Currently, both of these methods of glycolysis find industrial applications due to the fact that they can be used in chemical recycling of foams from industrial products, i.e., car seats [20] or refrigerators [21,22,23]. Glycerolysis is a modification of glycolysis, where glycerol is used as a depolymerizing agent. This type of chemical recycling of polyurethanes, similarly to glycolysis, can be realized with the mass excess of depolymerizing agent or depolymerized polyurethane.

Chemical recycling products obtained with mass excess of depolymerized polyurethane can be directly used in the synthesis of new polyurethane elastomers [24]. The application proposed by us involves using them as a modifier of natural rubber (NR) mixtures and vulcanizates properties [25]. In the literature, only a few urethane group-containing modifiers were proposed for rubber materials (for example, macromolecular hindered phenol antioxidants containing thioether and urethane groups [26]). Chemical recycling products obtained with the mass excess of depolymerizing agent results in recovery of polyol that can be used for the synthesis of polyurethane foams [3,27].

In accordance with the literature, the modification of the properties of rubber mixtures and vulcanizates can be realized by the addition of bio-based functional additives such as antioxidants or plasticizers. Examples of antioxidants proposed for use in rubber materials include: δ-tocopherol [28], cardanol [29], lignin [30,31], rambutan peel extract (which includes geraniin, corilagin, ellagic acid and gallic acid) [32], and oil palm leaf extract (*Elaeis guineensis*) [33]. Examples of bio-based plasticizers for rubber compounds and vulcanizates include cardanol [34], D-limonene and canola oil [35], palm oil [36], linseed oil [37], polymerized soybean oil [38], soybean oil modified by reaction with sulfur [39], and olive and orange oil [40].

The application of recycling products in rubber materials is mainly connected with using ground tire rubber (unmodified, compatibilized or reclaimed) for the preparation of rubber materials [41,42,43,44,45], or using oil from tire pyrolysis as a plasticizer for rubber compounds [46,47,48].

In our previous work [25], we studied the effect of polyurethane glycolysate on the chemical structure and properties of natural rubber/carbon black (NR/CB) composites. The glycolysate was synthesized by the reaction of polyurethane foam (PF) and 1,4-butanediol (BDO) as a depolymerizing agent. Glycolysis was conducted at 200 °C for 2 h using potassium acetate (CH_3_COOK) as a catalyst. The ratio of PF:BDO was equal to 6:1. The results were compared with the results for vulcanizates obtained using a commercial plasticizer, Nytex 810, which is a medium viscosity, hydrotreated naphthenic oil. It was found that the applied chemical recycling product affects the kinetics of the vulcanization of natural rubber mixtures, i.e., increasing the amount of glycolysate caused a decrease in scorch time, optimal vulcanization time, and curing rate index. Moreover, the applied modifier (i.e., polyurethane foam glycolysate) acted as an antidegradant. Higher retention of mechanical properties (i.e., tensile strength and elongation at break) during accelerated thermal aging in air was observed for natural rubber vulcanizates obtained using glycolysate.

The main aim of this study was to examine the effect of polyurethane glycerolysate on the selected properties of natural rubber mixtures and vulcanizates. The influence of the prepared chemical recycling product on rubber mixture vulcanization kinetics and swelling, and on the thermo-mechanical and mechanical properties of rubber vulcanizates, was studied. The prepared materials were subjected to accelerated thermal aging in air to estimate the possibility of using polyurethane glycerolysate as an antidegradant in natural rubber-based vulcanizates.

## 2. Materials and Methods

### 2.1. Materials

The natural rubber, accelerator T (tetramethylthiuram disulfide, TMTD), and sulfur were supplied by Guma Pomorska (Słupsk, Poland). Stearic acid, zinc oxide, sulfur and toluene were supplied by CHEMPUR (Piekary Śląskie, Poland). The polyurethane glycerolysate used as a modifier is our own product prepared at Gdańsk University of Technology (Gdańsk, Poland).

### 2.2. Preparation of Polyurethane Glycerolysate

**Figure 1:**
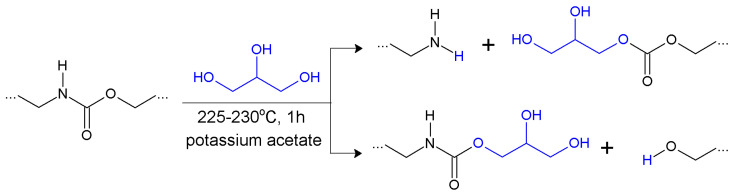
Schematic reaction of polyurethane glycerolysis.

The glycerolysate, used in this study as a modifier, was obtained by glycerolysis of cast poly(ester-urethane) using glycerol and potassium acetate (CH_3_COOK) as a catalyst. The ratio by mass of cast polyurethane to glycerol was equal to 4:1. The polyurethane was cut into small pieces and dosed in small portions for 1 h in glycerol heated to 230 °C under interrupted mixing. The reaction was continued at 225–230 °C for 1 h (after completed dosing of polyurethane). The obtained glycerolysate was a brown liquid characterized by hydroxyl number 332 mg KOH/g, viscosity 1.88 Pas at 50 °C, and density 1.19 g/cm^3^ at 25 °C. The simplified scheme of the glycerolysis is presented in Figure 1.

### 2.3. Preparation of Rubber Mixtures and Rubber Vulcanizates

The rubber mixtures were prepared using the components listed in the formulations presented in Table 1. The mixing process was realized using a laboratory two roll mill for about 15 min. The addition of each component was preceded by homogenous distribution of the previous added component to a natural rubber-based mixture. Vulcanization was realized by compression moulding at 150 °C with a pressure around 5 MPa. The optimum time to vulcanization was chosen based on vulcametric measurements and ranged from 8 to 10 min. Natural rubber mixtures are coded as NRM-XX, while vulcanizates are coded as NR-XX.

### 2.4. Testing Methods

The kinetics of rubber mixture vulcanization were characterized using a Monsanto R100S rheometer with oscillating rotor according to ISO 3417 [49]. The measurements were performed at 150 °C, which was the vulcanization temperature. The measurements obtained allow determination of the scorch time (*t*_2_), optimal vulcanization time (*t*_90_), minimum (ML) and maximum (MH) torque, and difference between them (ΔM).

Cure rate index (CRI) was calculated using the following equation:

(1) $$CRI=\frac{100}{t_{90}-t_{s2}}$$

The percentage reversion degree after 300 s from the time of reaching maximum value of torque (*R*_300_) was calculated using the following equation:

(2) $$R_{300}=\frac{M_{H}-M_{300}}{M_{H}}\cdot 100\%$$

where *M*_300_ is torque estimated at 300 s after occurrence of maximum torque.

The swelling properties of prepared natural rubber-based vulcanizates were characterized using equilibrium swelling in toluene at room temperature. The conducted tests allow determination of the swelling ratio (SR), which was calculated as the ratio of solvent mass absorbed by the sample (calculated as the difference between the mass of the swollen sample (*m*_0_) and the initial mass of the sample) to the mass of the sample before immersion in toluene (*m*_0_):

(3) $$SR=\frac{m_{s}-m_{0}}{m_{0}}\cdot 100\%$$

The cross-link density (*υ*) and molecular weight of rubber chains between cross-links (*M_c_*) were calculated using the following Flory-Rehner relationship:

(4) $$\nu =\frac{-\ln\left(1-V_{r}\right)-V_{r}-\chi \cdot V_{r}^{2}}{V_{s}\cdot \left(V_{r}^{\frac{1}{3}}-\frac{1}{2}\cdot V_{r}\right)}=\frac{1}{M_{c}}$$

where χ is the Flory-Huggins natural rubber-toluene interaction constant (χ = 0.38), *V_s_* is the molar volume of toluene (*V_s_* = 106.52 cm^3^/mol), and *V_r_* is the volume fraction of the natural rubber network in the swollen phase.

The volume fraction of the rubber network in the swollen phase (*V_r_*) is the ratio of the volume of rubber in the swollen sample (*V_rf_*) to the sum of the volume of rubber (*V_rf_*) and volume of toluene (*V_sf_*) in the swollen sample:

(5) $$V_{r}=\frac{V_{rf}}{V_{rf}+V_{sf}}=\frac{\frac{m_{rf}}{\rho_{r}}}{\frac{m_{rf}}{\rho_{r}}+\frac{m_{sf}}{\rho_{s}}}$$

where *m_rf_* is the mass of the rubber in the swollen sample, *m_sf_* is the mass of toluene in the swollen sample, *ρ_r_* is the density of natural rubber (*ρ_r_* = 0.9125 g/cm^3^), and *ρ_s_* is the density of toluene (*ρ_s_* = 0.867 g/cm^3^). The full test procedure is presented in our earlier work [50]. The results presented in this work are averages from three independent tests.

Dynamic mechanical thermal analysis (DMTA) was carried out using a DMA Q800 analyser (TA Instruments, New Castle, DE, USA) according to ISO 6721-1 [51]. The measurements were performed in a temperature range from −100 to +100 °C with a heating rate of 4 °C min^−1^ under nitrogen atmosphere. The test was carried out in bending mode at an operating frequency of 1 Hz. The dimensions of the samples for the test were 10 mm × 40 mm × 2 mm. The temperature dependence of storage modulus (E′), loss modulus (E″), and tangent delta (tan δ) was determined.

Thermogravimetric analysis (TGA) was carried out using a Pyris 1 analyser (Perkin Elmer, Waltham, MA, USA). The measurements were carried out in a temperature range from 50 to 600 °C with a heating rate of 20 °C min^−1^ under nitrogen atmosphere. The sample mass was around 10 mg.

Static tensile tests were carried out using a universal testing machine Zwick/Roell Z020 (Zwick, Ulm, Germany) according to ISO 37 [52] and standard dumbbell shape samples (characterized by average width and thickness in the gauge section equal to 5 and 2 mm, respectively). The tensile speed was equal to 300 mm min^−1^. Tensile properties including tensile strength (TS_b_); elongation at break (ε_b_); modulus at 100% (M100), 200% (M200), 300% (M300), 400% (M400), 500% (M500) and 600% (M600) elongation; and permanent elongation after break (ε_t_) were determined. All listed parameters are presented as averages from five independent measurements.

Hardness (H) was measured using a Shore type A durometer (Zwick, Germany) according to ISO 868 [53], while rebound resilience (RR) was determined using a Schob type rebound tester according to ISO 4662 [54]. The results of H and RR measurements presented in this study are averages of five independent tests.

### 2.5. Accelerated Thermal Aging of Natural Rubber Vulcanizates

Accelerated thermal aging in air at 70 °C for 7 and 14 days was conducted to assess the possibility of polyurethane glycerolysate acting as an antidegradant in natural rubber-based materials. The samples, prepared in standard dumbbell shape for static tensile test and rectangle shape for DMTA analysis, were aged in a laboratory oven with forced air circulation. A similar procedure of accelerated thermal aging can be found in the literature [55]. It should be pointed out that Nellen and Sellers [56] found that accelerated thermal aging in air of tread and carcass vulcanizates for 4 and 8 days at 70 °C corresponds to natural aging during 1 and 2 years, respectively. Ngolemasango, Bennett and Clarke [57] studied the effect of the aging temperature (in a range from 70 to 110 °C) on the tensile properties of natural rubber-based composites. Those authors found that extrapolation of in-service aging behavior from high temperature aging data (above 80 °C) can result in incorrect predictions. A similar conclusion, that lower temperatures better correlate with natural aging, was stated by Ahagon, Kida and Kaidou [58] in the case of aging of selected parts in heavy-duty tires.

## 3. Results and Discussion

### 3.1. Vulcanization Kinetics

**Figure 2:**
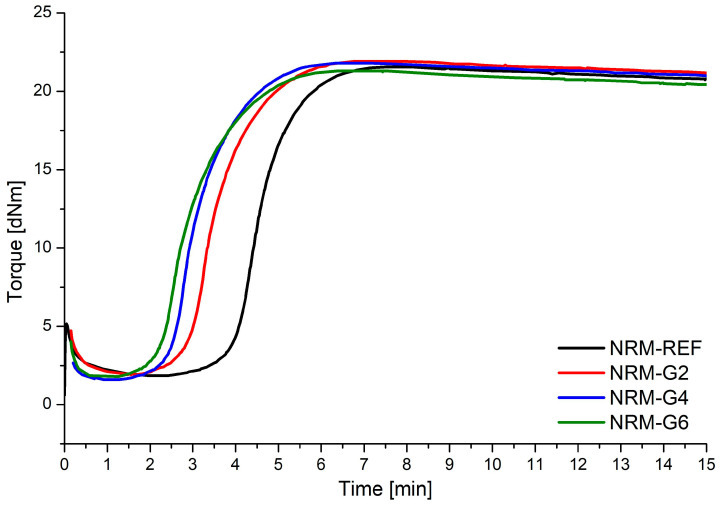
Vulcametric curves of prepared natural rubber mixtures.

The parameters characterizing the kinetics of the vulcanization process of prepared natural rubber mixtures are presented in Table 2 and supported by vulcametric curves presented in Figure 2. The addition of polyurethane glycerolysate in the rubber mixtures did not significantly affect the values of minimum and maximum torque or the difference between them. The difference between minimum and maximum torque is connected with the cross-link density of final vulcanizates, and it can be stated that the highest cross-link density can be obtained for NR-G4. It is also clearly visible that addition of polyurethane glycerolysate results in a decrease in scorch and optimum vulcanization time, while curing rate index decreases at the same time. The observed trend suggests that polyurethane glycerolysate promotes activation of the vulcanization process and acts as a vulcanization retardant. The degree of reversion of the vulcanization process (R300) is around 2% for all prepared materials.

### 3.2. Equilibrium Swelling and Cross-Linking Density

The swelling properties of natural rubber vulcanizates prepared using polyurethane glycerolysate as a modifier are summarized in Table 3. Increasing amounts of modifier results in an increase in swelling ratio (a higher mass of toluene was absorbed by the sample) as a result of decreasing cross-link density values (increasing of the molecular weight of chains between cross-links). It was found that in all investigated samples, cross-link density increased during the first seven days of accelerated thermal aging, while in the next seven days cross-link density decreased to below starting values (non-aged samples) for NR-REF and NR-G2, or slightly lower than those observed after the first 7 days of aging for NR-G4 and NR-G6. The increase in cross-link density is related to the post-curing effect (i.e., continuation of vulcanization) during accelerated thermal aging, which was reported for NR-based materials vulcanized using conventional systems [59]. The post-curing effect was also observed in our previous work for natural rubber vulcanizates prepared using polyurethane glycolysate as a modifier [25]. When the cross-link density reaches critical value, or degradation rate is higher than curing rate, a decrease in cross-link density is observed, as in our work. It should be pointed out that the applied modifier, i.e., polyurethane glycerolysate, acts as an antidegradant; with higher amounts of the modifier (more than 2 phr) the decrease in cross-link density is much lower than is the case for the reference sample (NR-REF), where the final value is lower than for non-aged materials.

### 3.3. Thermo-Mechanical Properties

**Figure 3:**
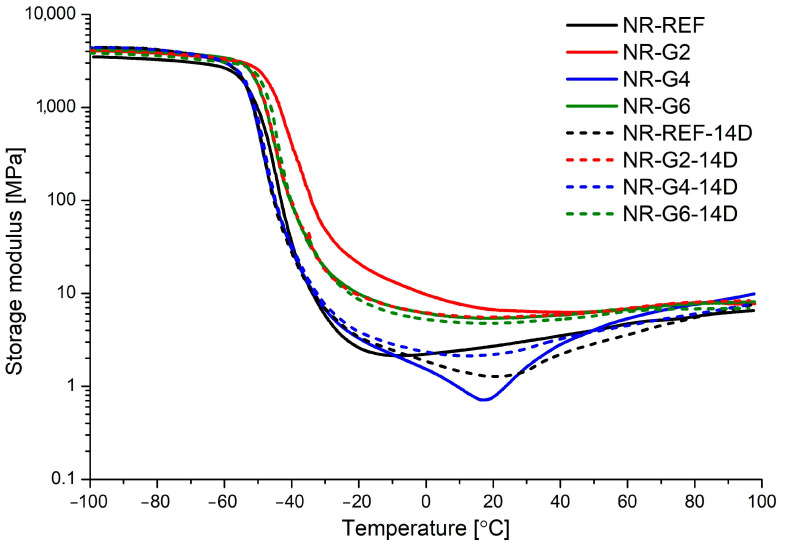
Storage modulus versus temperature for natural rubber vulcanizates non-aged (NR-REF, NR-G2, NR-G4 and NR-G6) and subjected to accelerated thermal aging in air (NR-REF-14D, NR-G2-14D, NR-G4-14D and NR-G6-14D).

**Figure 4:**
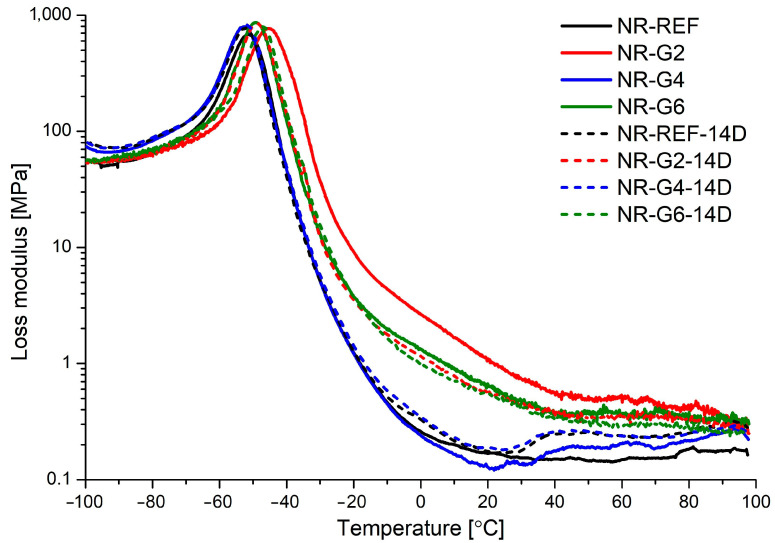
Loss modulus versus temperature for natural rubber vulcanizates non-aged (NR-REF, NR-G2, NR-G4 and NR-G6) and subjected to accelerated thermal aging in air (NR-REF-14D, NR-G2-14D, NR-G4-14D and NR-G6-14D).

**Figure 5:**
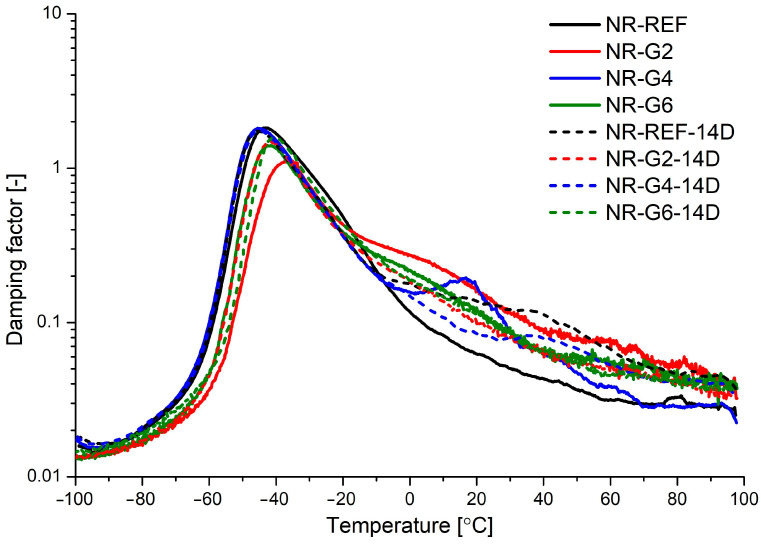
Damping factor versus temperature for natural rubber vulcanizates non-aged (NR-REF, NR-G2, NR-G4 and NR-G6) and subjected to accelerated thermal aging in air (NR-REF-14D, NR-G2-14D, NR-G4-14D and NR-G6-14D).

Viscoelastic properties determined by DMTA for prepared NR-based vulcanizates (non-aged and subjected to accelerated thermal aging in air (14 days, 70 °C)) are summarized in Table 4. Temperature dependence of storage modulus (E′), loss modulus (E″), and damping factor are presented in Figure 3, Figure 4 and Figure 5, respectively.

Storage modulus (E′) is related to the stiffness of the materials, so the highest values of this parameter are observed in the glassy state. The addition of polyurethane glycerolysate results in an increase in E′ in the glassy state, and the observed reinforcing effect is probably caused by the presence of modifier which becomes rigid at low temperature in the rubber network. In the main chains of glycerolysate polar moieties are present, i.e., urethane and hydroxyl, which interact with each other by hydrogen bonds. The reinforcing effect may also result from the formation of interpenetrating polymer networks between the natural rubber chains and oligomeric polyurethane glycerolysate.

The glass transition results in improved polymer chain mobility, and increasing energy dissipation during molecular rearrangements is visible as increasing loss modulus (E″) during the glass transition. These molecular rearrangements lead to non-reversible deformations [60]. In our work, increasing loss modulus at glass transition is connected with a higher ratio of polyurethane glycerolysate to natural rubber mixture, and it may result from a plasticizing effect introduced by the oligomeric modifier.

The damping factor (tanδ) is defined as the ratio of the loss modulus (E″) to the storage modulus (E′), and glass transition temperature (T_g_) is defined as the temperature at which the damping factor reaches its maximum value. The damping factor is related to the ability of polymer to dissipate the energy of deformation. Increasing amounts of polyurethane glycerolysate and subjection of the materials to accelerated thermal aging caused a decrease in damping factor value at T_g_. The observed trends suggest a decrease in damping properties of the obtained materials. The decrease of tanδ values during thermal aging for samples containing polyurethane glycerolysate is less marked.

Increasing amounts of added modifier results in a decrease in glass transition temperature, which suggests that introduction of polyurethane glycerolysate in the rubber matrix results in increasing polymer chain mobility. This may result from the nature of polyurethane glycerolysate, which can act as a plasticizer, or a decrease in cross-linking density caused by the addition of the mentioned modifier. The accelerated aging (14 days, 70 °C, air) of obtained vulcanizates caused an increase in glass transition temperature, which suggests that a greater restriction of polymer chain movements occurs. This may be connected with higher cross-linking density resulting from the continuation of rubber cross-linking during the accelerated thermal aging, and this trend is reported in the literature as a post-curing effect [59]. It should also be noted that an increase in T_g_ is connected with increasing storage modulus at the rubbery state (E′ at 21 °C).

### 3.4. Thermal Stability

**Figure 6:**
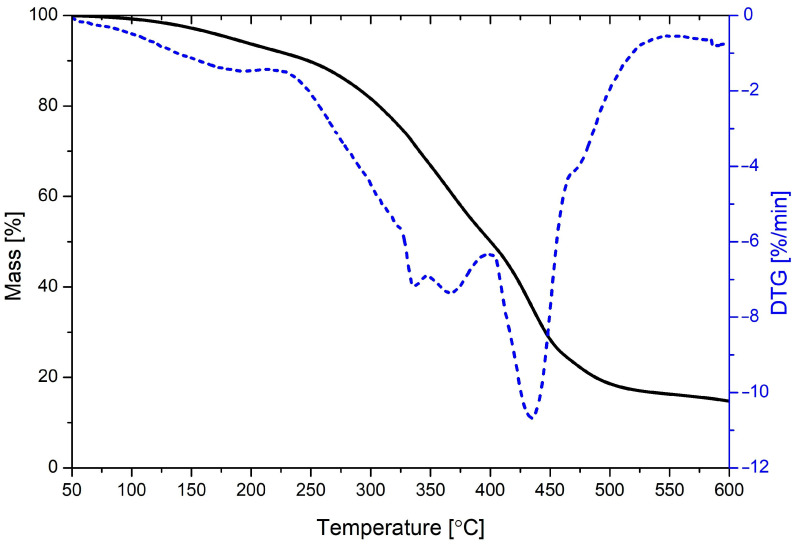
TG and DTG curves for polyurethane glycerolysate.

**Figure 7:**
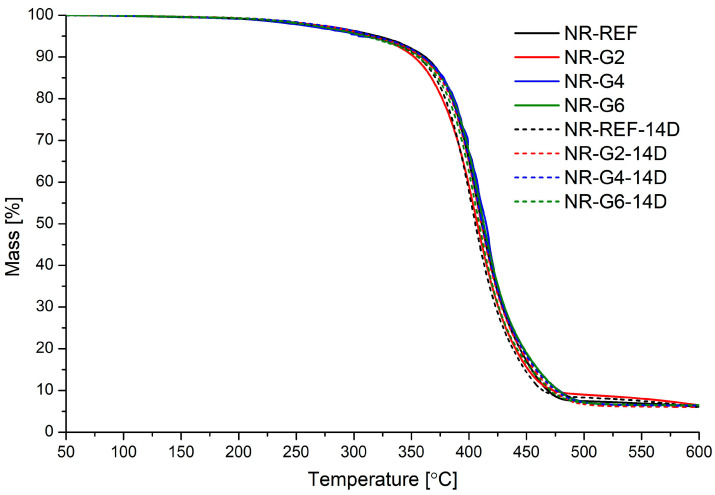
TG curves for prepared natural rubber vulcanizates.

**Figure 8:**
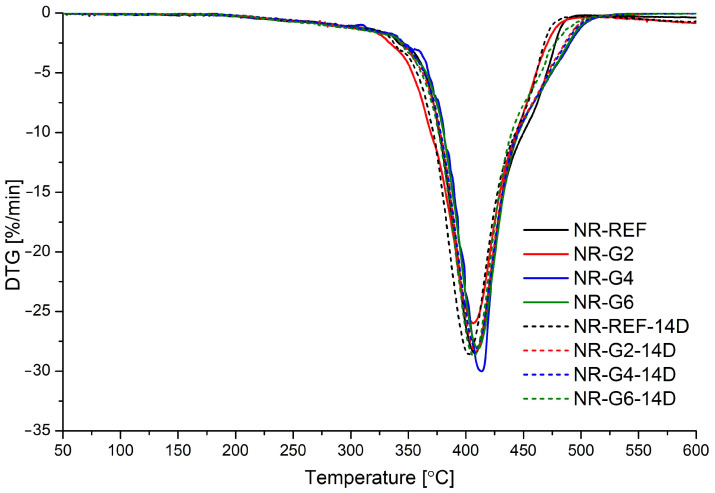
DTG curves for prepared natural rubber vulcanizates.

Parameters characterizing the thermal stability of glycerolysate and obtained vulcanizates, i.e., temperatures at which 5 and 10 wt.% mass loss occurs (T_d5_ and T_d10_), and the temperature at which mass loss occurs with the highest speed (T_d max_), are presented in Table 5. The presented parameters are supported by thermogravimetric (TG) and derivative thermogravimetric (DTG) curves. The TG and DTG curve for glycerolysate is presented in Figure 6, while TG and DTG curves for prepared rubber vulcanizates are presented in Figure 7 and Figure 8, respectively.

Thermal decomposition of glycerolysate is a complex process (consisting of at least three steps) which involves decomposition of urethanes and ester moieties present in the main chains of this oligomeric product. Thermal decomposition of prepared natural rubber vulcanizates is a one-step process which involves mainly decomposition of cis-1,4-polyisoprene connected with release of isoprene and other products (for example, limonene). The addition of polyurethane glycerolysate did not significantly affect the thermal stability of prepared natural rubber vulcanizates. The determined parameters (T_d5_, T_d10_ and T_d max_) are very similar, including after accelerated thermal aging of vulcanizates.

### 3.5. Mechanical Properties

Static tensile properties of NR-based vulcanizates prepared with polyurethane glycerolysate as a modifier are presented in Table 6. The addition of chemical recycling product to rubber mixtures results in vulcanizates with slightly higher tensile strength and moduli at selected elongations. Unfortunately, presence of polyurethane glycerolysate in rubber vulcanizates results in an increase in permanent elongation after break, which is caused by the plasticizing effect of the applied modifier (oligomeric product). It is also clearly visible that polyurethane glycerolysate improves stability of mechanical properties during accelerated thermal aging of vulcanizates, especially in comparison to reference vulcanizates (prepared with modifier). It should also be pointed out that for the reference sample, significant decrease in tensile properties (i.e., tensile strength and elongation at break) occurred after 14 days of accelerated thermal aging in air. For example, tensile strength is more than ten times lower than the initial value (before accelerated thermal aging). Increasing the amount of added chemical recycling product results in higher values of tensile strength and elongation at break after 7 and 14 days of aging in air at 70 °C. This observation suggests that the polyurethane glycerolysate can probably act as an antidegradant in natural rubber due to the presence of urethane groups in the main chains of the oligomeric product. The presence of urethane moieties results from the realization of the glycerolysis process with mass excess of the depolymerized polyurethane (Figure 1). Similar trends were observed in our previous work [25] for the NR/CB composites prepared using polyurethane glycolysate (also obtained using mass excess of depolymerized polyurethane). It should also be noted that, according to the literature, macromolecular-hindered phenol containing thioether and urethane groups was proposed as an antioxidant for natural rubber [26].

The analysis of retention of tensile strength and elongation at break after thermal aging in air at 70 °C (Table 7) indicates that polyurethane glycerolysate acts as an antidegradant in natural rubber vulcanizates. Similar values of retention of TSb and ε_b_ (NR-G6, 14 days, 70 °C) was observed by us in natural rubber composites filled with carbon black and containing commercially available antioxidants, i.e., *N*-(1,3-dime-thylbutyl)-*N′*-phenyl-*p*-phenylenediamine (6PPD) and 2,2,4-trimethyl-1,2-dihydroquinoline (TMQ) [57]. In comparison to our previous work related to NR/CB composites modified by polyurethane glycolysate [25], the retention of TS_b_ and ε_b_ (6 phr of modifier, 14 days, 70 °C) was equal to 85.2% and 79.4%, respectively.

The hardness and rebound resilience of the obtained natural rubber vulcanizates is not significantly affected by addition of polyurethane glycerolysate (Table 8). The hardness values are in the range 44.8–45.7 ShA, while rebound resilience values are in the range 77.0–79.4%. The highest values of these parameters were obtained for NR-G2 vulcanizate characterized by a lower amount of added polyurethane glycerolysate.

## 4. Conclusions

Polyurethane glycerolysate, prepared in this work, is an oligomeric product obtained by the reaction of mass excess of polyurethane and glycerol. It can be used as a polyol substitute in the synthesis of new polyurethanes. In this study, we propose the use of polyurethane glycerolysate as a multifunctional modifier of natural rubber mixtures and vulcanizates. Polyurethane glycerolysate affects the kinetics of the vulcanization process. It was found that scorch time and optimal vulcanization time decreased. At the same time, curing rate index decreased, which suggests that the applied chemical recycling product promotes the activation of the vulcanization process and acts as a vulcanization retardant. Accelerated thermal aging in air confirms that polyurethane glycerolysate acts as an antidegradant. Increasing amounts of modifier result in higher stabilization of mechanical properties (i.e., tensile strength and elongation at break) in comparison to the reference sample (without any modifier). The roles of polyurethane glycerolysate are related to its chemical structure, i.e., oligomeric chains containing urethane moieties and terminating mainly in hydroxyl groups. The obtained results suggest that polyurethane glycerolysate can be a valuable functional additive for natural rubber-based mixtures and vulcanizates, which extends the range of possible applications of polyurethane chemical recycling products.

## Figures and Tables

**Table 1:** Formulations of prepared natural rubber mixtures.

Sample Code	NRM-REF	NRM-G2	NRM-G4	NRM-G6
natural rubber	100	100	100	100
stearic acid	3	3	3	3
zinc oxide	5	5	5	5
glycerolysate	-	2	4	6
accelerator T	0.5	0.5	0.5	0.5
sulfur	2	2	2	2

**Table 2:** Parameters characterizing the vulcanization process of prepared natural rubber mixtures.

Sample Code	T	ML	MH	ΔM	t_s2_	t_90_	CRI	R300
	[°C]	[dNm]	[dNm]	[dNm]	[min]	[min]	[min^−1^]	[%]
NRM-REF	150	1.85	21.55	19.7	3.92	5.66	57.5	2.3
NRM-GR2	150	1.91	21.92	20.01	2.85	4.91	48.5	2.0
NRM-GR4	150	1.60	21.80	20.20	2.48	4.48	50.0	2.0
NRM-GR6	150	1.79	21.30	19.51	2.24	4.46	45.0	2.4

**Table 3:** Swelling properties determined by equilibrium swelling in toluene at room temperature for prepared natural rubber vulcanizates.

Material Code	Aging Time	SR	V_r_	v·10^−1^	M_c_
	[Days]	[%]	[-]	[mol/dm^3^]	[g/mol]
NR-REF	0	351.3 ± 3.4	0.21167 ± 0.00158	0.17514 ± 0.00301	5711 ± 99
	7	349.4 ± 2.4	0.21286 ± 0.00112	0.17741 ± 0.00216	5637 ± 69
	14	368.3 ± 3.8	0.20382 ± 0.00171	0.16061 ± 0.00306	6228 ± 120
NR-G2	0	342.5 ± 0.1	0.21269 ± 0.00006	0.17708 ± 0.00011	5647 ± 4
	7	326.7 ± 1.9	0.22083 ± 0.00095	0.19317 ± 0.00193	5177 ± 52
	14	350.3 ± 1.4	0.20890 ± 0.00066	0.16990 ± 0.00123	5886 ± 43
NR-G4	0	347.7 ± 2.4	0.20668 ± 0.00114	0.16580 ± 0.00209	6032 ± 76
	7	325.5 ± 1.5	0.21794 ± 0.00072	0.18735 ± 0.00144	5338 ± 41
	14	334.1 ± 1.4	0.21368 ± 0.00070	0.17898 ± 0.00136	5587 ± 42
NR-G6	0	356.5 ± 2.2	0.19920 ± 0.00098	0.15244 ± 0.00170	6560 ± 74
	7	336.4 ± 0.5	0.20897 ± 0.00024	0.17004 ± 0.00045	5881 ± 16
	14	336.0 ± 0.6	0.20940 ± 0.00030	0.17084 ± 0.00056	5853 ± 19

**Table 4:** Parameters characterizing thermo-mechanical properties of obtained vulcanizates.

Material	Aging Time	E′_max_	E′ @ 21 °C	E″_max_	T_E″max_	T_g_	tanδ @ T_g_
	[Days]	[MPa]	[MPa]	[MPa]	[°C]	[°C]	[-]
NR-REF	0	3500	2.73	683	−51.5	−42.8	1.820
	14	4074	6.61	768	−45.7	−35.8	1.122
NR-G2	0	4278	0.81	797	−52.8	−45.5	1.809
	14	4188	5.42	863	−49.1	−42.5	1.406
NR-G4	0	4426	1.27	780	−52.3	−45.6	1.743
	14	4060	5.55	850	−49.0	−41.5	1.486
NR-G6	0	4425	2.22	832	−52.4	−44.7	1.751
	14	3847	4.76	797	−47.2	−40.3	1.585

**Table 5:** Parameters characterizing thermal stability of glycerolysate and obtained vulcanizates.

Material	Aging Time	T_d5_	T_d10_	T_d max_	Char Yield at 600 °C
	[Days]	[°C]	[°C]	[°C]	[%]
GLYCEROLYSATE	-	182.6	247.6	335.6/434.8	14.8
NR-REF	0	320.6	362.0	407.8	6.1
	14	315.1	355.7	402.6	6.0
NR-G2	0	313.7	352.4	406.4	6.4
	14	316.2	358.6	408.3	6.1
NR-G4	0	304.0	358.8	413.6	6.4
	14	318.1	360.5	408.4	6.4
NR-G6	0	313.4	358.4	410.7	6.5
	14	309.6	355.6	406.7	6.4

**Table 6:** Tensile properties of prepared natural rubber vulcanizates.

Material	Aging Time	TS_b_	ε_b_	M100	M200	M300	M400	M500	M600	ε_t_
	[Days]	[MPa]	[%]	[MPa]	[MPa]	[MPa]	[MPa]	[MPa]	[MPa]	[%]
NR-REF	0	21.0 ± 2.6	647 ± 37	0.86	1.44	2.31	3.92	7.87	16.13	8.6 ± 1.2
	7	17.5 ± 0.9	648 ± 38	0.84	1.43	2.28	3.80	7.25	14.46	7.9 ± 1.1
	14	1.8 ± 0.4	268 ± 56	0.76	1.32	2.14	-	-	-	0.6 ± 0.5
NR-G2	0	22.5 ± 0.8	651 ± 8	0.97	1.55	2.30	3.56	6.95	15.10	14.3 ± 1.5
	7	19.5 ± 1.7	543 ± 22	1.02	1.83	3.17	6.16	14.21	-	12.4 ± 2.2
	14	12.1 ± 1.5	482 ± 18	0.90	1.72	3.18	6.57	-	-	5.4 ± 1.9
NR-G4	0	21.9 ± 0.3	609 ± 19	0.88	1.50	2.39	4.29	10.36	21.63	15.3 ± 3.7
	7	20.2 ± 0.2	571 ± 20	0.96	1.78	3.15	6.24	14.45	-	10.9 ± 1.4
	14	11.2 ± 0.7	495 ± 5	0.84	1.55	2.72	5.17	-	-	3.3 ± 0.2
NR-G6	0	23.1± 0.9	638 ± 9	0.81	1.40	2.25	4.05	9.75	20.57	14.3 ± 0.8
	7	18.8 ± 1.7	607 ± 7	0.89	1.50	2.41	4.05	8.12	16.72	10.6 ± 1.3
	14	16.8 ± 0.5	556 ± 18	0.86	1.61	2.90	5.70	12.71	-	5.5 ± 1.4

**Table 7:** Retention of selected tensile properties during accelerated thermal aging.

Material	Aging Time	Retention of TS_b_	Retention of ε_b_
	[Days]	[%]	[%]
NR-REF	7	83.3	100
	14	8.6	41.4
NR-G2	7	86.7	83.4
	14	53.8	74.0
NR-G4	7	92.2	93.8
	14	51.1	81.3
NR-G6	7	81.4	95.1
	14	72.7	87.1

**Table 8:** Hardness and rebound resilience of prepared natural rubber-based vulcanizates.

Material	Aging Time	Hardness	Rebound Resilience
	[Days]	H [°Sh A]	RR [%]
NR-REF	0	44.8 ± 0.1	77.0 ± 0.2
	7	42.1 ± 0.3	73.4 ± 0.6
	14	41.7 ± 0.2	73.1 ± 0.4
NR-G2	0	45.7 ± 0.5	79.4 ± 0.9
	7	43.5 ± 0.2	75.9 ± 0.5
	14	44.8 ± 0.1	74.8 ± 0.5
NR-G4	0	45.2 ± 0.2	78.6 ± 0.7
	7	43.4 ± 0.2	78.2 ± 0.4
	14	44.4 ± 0.3	75.5 ± 0.7
NR-G6	0	44.8 ± 0.4	78.7 ± 0.6
	7	43.5 ± 0.2	77.3 ± 0.8
	14	44.1 ± 0.2	75.9 ± 0.4

## Data Availability

Data are contained within the article.
